# Inhibition of Stat3 signaling ameliorates atrophy of the soleus muscles in mice lacking the vitamin D receptor

**DOI:** 10.1186/s13395-017-0121-2

**Published:** 2017-01-25

**Authors:** Suchitra D. Gopinath

**Affiliations:** Translational Health Science and Technology Institute (THSTI), NCR Biotech Science Cluster, 3rd Milestone, Faridabad-Gurgaon Expressway, PO box #04, Faridabad, 121001 India

**Keywords:** Atrophy, Soleus, Tibialis anterior, Myostatin, p-70S6K, p-rpS6, p-Stat3, Satellite cells, Skeletal muscle

## Abstract

**Background:**

Although skeletal muscle wasting has long been observed as a clinical outcome of impaired vitamin D signaling, precise molecular mechanisms that mediate the loss of muscle mass in the absence of vitamin D signaling are less clear. To determine the molecular consequences of vitamin D signaling, we analyzed the role of signal transducer and activator of transcription 3 (Stat3) signaling, a known contributor to various muscle wasting pathologies, in skeletal muscles.

**Methods:**

We isolated soleus (slow) and tibialis anterior (fast) muscles from mice lacking the vitamin D receptor (VDR^−/−^) and used western blot analysis, quantitative RTPCR, and pharmacological intervention to analyze muscle atrophy in VDR^−/−^ mice.

**Results:**

We found that slow and fast subsets of muscles of the VDR^−/−^ mice displayed elevated levels of phosphorylated Stat3 accompanied by an increase in Myostatin expression and signaling. Consequently, we observed reduced activity of mammalian target of rapamycin (mTOR) signaling components, ribosomal S6 kinase (p70S6K) and ribosomal S6 protein (rpS6), that regulate protein synthesis and cell size, respectively. Concomitantly, we observed an increase in atrophy regulators and a block in autophagic gene expression. An examination of the upstream regulation of Stat3 levels in VDR^−/−^ muscles revealed an increase in IL-6 protein expression in the soleus, but not in the tibialis anterior muscles. To investigate the involvement of satellite cells (SCs) in atrophy in VDR^−/−^ mice, we found that there was no significant deficit in SC numbers in VDR^−/−^ muscles compared to the wild type. Unlike its expression within VDR^−/−^ fibers, *Myostatin* levels in VDR^−/−^ SCs from bulk muscles were similar to those of wild type. However, VDR^−/−^ SCs induced to differentiate in culture displayed increased p-Stat3 signaling and *Myostatin* expression. Finally, VDR^−/−^ mice injected with a Stat3 inhibitor displayed reduced Myostatin expression and function and restored active p70S6K and rpS6 levels, resulting in an amelioration of loss of muscle mass in the soleus muscles.

**Conclusions:**

The loss of muscle mass in slow muscles in the absence of vitamin D signaling is due to elevated levels of phosphorylated Stat3 that leads to an increase in Myostatin signaling, which in turn decreases protein synthesis and fiber size through the phosphorylation of p70S6K and rpS6, respectively.

**Electronic supplementary material:**

The online version of this article (doi:10.1186/s13395-017-0121-2) contains supplementary material, which is available to authorized users.

## Background

Vitamin D deficiency has only recently been recognized as a worldwide problem and has wide-ranging effects besides skeletal defects [1–3]. Several tissues are indirectly affected by impaired vitamin D signaling due to perturbations in calcium and phosphate-dependent signaling processes [4, 5]. In the skeletal muscle, vitamin D regulation of calcium uptake modulates glucose metabolism and transcriptional upregulation of myocyte-enhancer factors 2A and 2D [6, 7]. Consequently, patients with genetic defects in the vitamin D-activating enzyme, 25-hydroxyvitamin D 1α-hyroxylase, and in heterogeneous loss of function mutations in the vitamin D receptor (VDR) display progressive muscle weakness and muscle atrophy [8–10]. However, initial evidence for the direct action of vitamin D on the skeletal muscle came from studies in chicks demonstrating that there was no correlation between myopathy as a result of vitamin D deficiency and phosphate and calcium levels in serum and that the restoration of calcium levels did not alleviate muscle weakness [11]. These results were further corroborated in experiments examining abnormalities in skeletal muscle development in mutant mice lacking VDR (VDR^−/−^) that manifested in a manner independent of changes in serum metabolites [12, 13]. However, signaling pathways that link the aberrations in myogenic gene expression and the progressive muscle atrophy observed postnatally in VDR^−/−^ muscles have been unexplored. More importantly, recent evidence indicates that components of the vitamin D signaling cascade are expressed in human precursor cells and that the VDR in rodent quadriceps muscle responds to 1α,25(OH)_2_D_3_ treatment [14, 15]. Nevertheless, mechanistic studies that account for the muscle atrophy observed in vitamin D-deficient individuals are lacking.

The process of muscle wasting is an inherently heterogeneous process, with different sets of muscles consisting of distinct fiber types displaying differential sensitivities to the same stimulus [16–19]. In humans, vitamin D-deficient patients displayed a greater tendency toward type IIb fiber (fast twitch) atrophy than type 1 fiber (slow twitch) atrophy [20]. However, in the VDR^−/−^ mice, muscle fibers are smaller than wild type irrespective of the fiber type [13]. These differences in the effects of vitamin D signaling between mice and humans suggest that the effects of VDR deletion are more multifarious than the deficiency of 1α,25(OH)_2_D_3_ alone. As such, since both subsets are impacted by the loss of vitamin D signaling, the VDR^−/−^ mice offer a model that allows us to address the consequences of specific signaling pathways in promoting muscle wasting in slow and fast subsets of muscles. More importantly, mechanistic insights in the VDR^−/−^ mouse model provide targets for intervention in cases where supplementation of vitamin D is not a viable option, such as in the instances of genetic defects resulting in loss of function mutations in the vitamin D signaling system.

Persistent signal transducer and activator of transcription 3 (Stat3) signaling has been linked to muscle wasting in response to enhanced interleukin-6 (IL-6) stimulation through Myostatin [21, 22]. In this study, we examine the role of activated Stat3 signaling in promoting skeletal muscle atrophy observed in VDR^−/−^ mice, in muscles with a predominance of type 1 fibers (soleus) and type II fibers (tibialis anterior (TA)). We show that increased Stat3 signaling is accompanied by an increase in the expression of *Myostatin*, a negative regulator of muscle mass. Consequently, Myostatin signaling through p-Smad3 reduces phosphorylated ribosomal protein S6 kinase (p-p70S6K) and its substrate, phosphorylated ribosomal protein S6 (p-rpS6), that represent hallmarks of mammalian target of rapamycin (mTOR) signaling [23]. While p70S6K activity is associated with modulating protein synthesis, rpS6 phosphorylation has been established as a key determinant of cell proliferation, cell size, and glucose homeostasis [24, 25]. Finally, to determine the importance of the Stat3 pathway in mediating the effects of vitamin D on muscle mass, we address whether pharmacological inhibition of Stat3 signaling in vivo can ameliorate the loss of muscle mass observed in the absence of vitamin D signaling.

## Methods

### Animals

VDR-null mutant mice were obtained from Jackson Laboratories (stock no: 006133, B6.129S4-Vdr^tm1Mbd^ lJ; Bar harbor, ME, USA) and bred at the Small Animal Facility at the National Institute of Immunology, New Delhi, India. All mice were fed ad libitum with a commercially available rodent chow and with tap water supplemented with 1% calcium, 1% phosphate, and 2% lactose. Mice were housed in a sterile facility in individually ventilated cages (IVC). Animal strain maintenance, post mortem collection of tissues, drug treatments, and husbandry were carried out according to the guidelines of and with the approval of the Animal Ethics Committee at the National Institute of Immunology.

### RNA extraction and quantitative RT-PCR

Dissected soleus and TA muscles were snap frozen in liquid nitrogen and homogenized in TRIzol*®* Reagent (1 ml/mg tissue; Thermo Fisher Scientific) to isolate total muscle RNA as per the manufacturer’s recommendations. Total RNA was quantified with a Nanodrop 8000 Spectrophotometer (Thermo Scientific, Wilmington) and a ratio of 2 for the absorbance of 260 to 280 nm was used to determine the RNA quality. First-strand cDNA was synthesized from total RNA using the First Strand SuperScript Synthesis System with SuperScript II reverse transcriptase according to the manufacturer’s protocols (Invitrogen, Carlsbad, CA). Quantitative RT-PCR was performed using the Mastercycler® RealPlex^2^ from Eppendorf with *Power* SYBR® Green PCR Master Mix (Applied Biosystems). Each sample was amplified in triplicates using primers specific to *Myostatin* [22], *Foxo3* [26], *Murf1* and *C/EBP δ* [22], *LC3b* and *Bnip3* [27], and glyceraldehyde 3-phosphate dehydrogenase (*Gapdh*) [26]. Expression levels were normalized to *Gapdh*.

### Satellite cell isolation and fluorescence-activated cell sorting

Satellite cells were isolated from the hind limb muscles as described earlier [28]. After enzymatic digestion of the myofibers, mononuclear cells were stained with Vcam-biotin (clone 429; BD Bioscience), CD31-APC (clone MEC 13.3; BD Bioscience), CD45-APC (clone 30-F11; BD Bioscience), and Sca-1-PE-cy7 (clone D7; eBioscience) [28, 29]. Streptavidin-PE was used to amplify the Vcam signal (BD Bioscience). Cells were sorted using a BD fluorescence-activated cell sorting (FACS) ARIA III cell sorter. A post-sort profile was carried out after every experiment to determine sort purity. Additionally, a small fraction of cells was stained for MyoD to determine the myogenic status of the sorted cells.

### Western blotting and antibodies

Protein extracts from the tibialis anterior and soleus muscles of wild-type and VDR^−/−^ mice were obtained by homogenizing muscles in lysis buffer (50 mM Tris-HCl pH 7.5, 150 mM NaCl, 5 mM EDTA, 1% NP40, 0.5% sodium deoxycholate, 0.1% SDS) containing phosphatase inhibitors (10 mM Na_3_VO_4_, 100 mM NaF) and protease inhibitors (Roche). The proteins were resolved by SDS-PAGE (8%) and transferred onto nitrocellulose membranes. The membranes were incubated with primary antibodies followed by incubation with HRP-conjugated anti-mouse or anti-rabbit secondary antibodies and visualized using Supersignal West Pico Chemiluminescent Substrate (Thermo Scientific, Rockford, IL, USA). Blots were probed with antibodies against p-Stat3 (clone # B-7, cat # sc-8059, Santa Cruz Biotechnology), p-rpS6 (clone # 91B2, cat # 4857), S6 ribosomal protein (clone # 5G10, cat # 2217), p-p70S6 kinase (Thre389, #9205, Cell Signaling Technology, Beverly, MA), p70S6 kinase (#9202s, Cell Signaling Technology, Beverly, MA), p-Stat5 (Clone C11C5, cat # 9359), Stat5 (cat # 9363), Stat3 (cat # 4904), IL-6 (cat # 12912, Cell Signaling Technology, Beverly, MA), p-Smad3 (Ser423/425) (C25A9, #9520, Cell Signaling Technology, Beverly, MA), slow myosin (NOQ 7.5.4D, cat # M8421, Sigma-Aldrich), Myogenin (F5D, #ab1835 Abcam), GAPDH, and β-actin (cat # sc-47724 and # sc-47778, respectively, Santa Cruz Biotechnology).

### Enzyme-linked immunosorbent assay

ELISA plates (Nunc MaxiSorp) were coated overnight at 4 °C with 1 μg of the respective capture antibody or mock BSA prepared in coating buffer as per the manufacturer’s instructions. After blocking in the ELISA/ELISPOT diluent, sera isolated from retro-orbital bleeds and muscle lysates from the soleus and TA muscles from wild-type (WT) and V mice were incubated for 3 h at 37 °C. Plates were thoroughly washed and incubated with the respective biotinylated goat polyclonal anti-mouse antibody against IL-6, interferon-γ (IFNγ) (cat # 88-7064-88 and cat # 88-734-88, respectively, eBioscience), and tumor necrosis-α (TNFα) (cat # 430904, BioLegend). Binding was detected by peroxidase linked to avidin. Enzyme activity was measured using TMB.

### Drug treatment

Four-and-a-half-week-old VDR^−/−^ male mice that were paired for comparable weights were injected with 6.25 mg/kg of the Stat3 inhibitor Xlll, C188-9 (Merck, Millipore, catalog # 573128-10MGCN) in 5% dextrose in water (5DW), or 5DW alone, intraperitoneally daily for 13 days. Wild-type mice were injected with the Stat3 inhibitor or 5DW as corresponding controls. On the 14th day, mice were euthanized and the soleus and TA muscles were collected and processed for protein and RNA isolation.

### Histological analysis

The soleus muscles harvested from the mice were snap frozen in liquid nitrogen and prepared for obtaining 8 μm cryosections. Histological analysis was performed using hematoxylin and eosin (Sigma-Aldrich) staining. A minimum of 800 fibers were examined in replicates of three for each group. The circumference of each fiber was outlined using ImageJ software (v. 1.49).

### Statistical analyses

A minimum of five replicates in terms of individual animals was used per experiment, and data are represented as mean ± SEM. Student’s unpaired *t* test with unequal variance was used to test for statistically significant differences between groups using GraphPad Prism Software (Version 5.0). The *p* < 0.05 level was considered significant. For isolation of RNA from SCs, a minimum of three animals was used and FACS-sorted cells were pooled for further analysis.

## Results

### Activated Stat3 protein (p-Stat3) is increased in VDR^−/−^ muscles

**Fig. 1:**
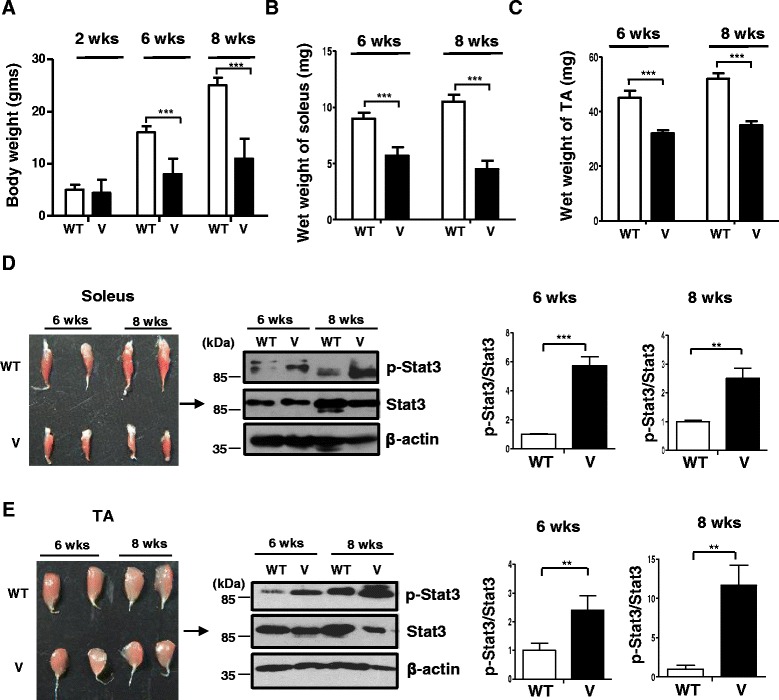
Reduced weight and elevated p-Stat3 levels displayed by VDR^−/−^ muscles. Graph shows average weights of the whole body of wild-type (*W*) and VDR^−/−^ mice (*V*) at 2, 6, and 8 weeks of age (**a**) of the soleus (**b**) and TA muscles (**c**) that were dissected out from WT and V mice at 6 and 8 weeks (*n* = 6) (**p < 0.01, ***p < 0.005). **d** *Left panel* shows dissected soleus muscles from wild-type (*WT*) (*top row*) and VDR^−/−^ mice (*V*) (*bottom row*) at 6 and 8 weeks of age. *Right panel* shows a representative western blot analysis of the lysates from the same subset of muscles from WT and V and probed for p-Stat3 antibody (*top panel*) and total Stat3 (*middle panel*). *Graphs to the right* show quantitative analyses of replicative blots of the ratio of relative intensities of p-Stat3 to total Stat3 at 6 and 8 weeks, respectively, between WT and V muscles (*n* = 6) (**p < 0.01). **e** *Left panel* shows dissected tibialis anterior (TA) muscles from WT (*top row*) and V mice (*bottom row*) at 6 weeks and 2 months of age. *Right panel* shows a representative western blot analysis of lysates from the same subset of muscles from WT and V and probed with antibodies as in **d**. *Graphs to the right* show similar analyses as **d** of ratios of p-Stat3 to total Stat3 at 6 and 8 weeks, respectively, between WT and V muscles (*n* = 6) (***p < 0.005). Total Stat3 levels were first normalized to β-actin that serves as a loading control prior to normalization of p-Stat3 to total Stat3

Previous reports have demonstrated that although the VDR^−/−^ mice display no defects in growth or difference in body weight before weaning (3 weeks), the following period is characterized by a significant reduction in body weight with a viability of a maximum of 15 weeks (Fig. 1a) [12]. Additionally, as earlier reported [30], there were no differences in the quantity of food intake between the wild-type and VDR^−/−^ mice (3 g ± 0.25/day) at the ages examined. In terms of activity, previous studies have reported profound muscular and motor impairments that affect locomotory behavior in VDR^−/−^ mice after 10 weeks of age [31]. Hence, we chose time points spanning through midlife to avoid secondary consequences from external confounding factors that contribute to the progressive nature of the abnormalities. While no differences in body weight could be observed at 2 weeks of age, both the soleus and TA muscles from VDR^−/−^ mice display reduced weight in comparison to age- and sex-matched wild-type littermate controls examined at 6 weeks and 2 months (Fig. 1b, c, respectively).

To test whether levels of p-Stat3 are elevated in VDR^−/−^ muscles, we isolated the soleus and TA muscles from wild-type and VDR^−/−^ mice that were 6 and 8 weeks old. Western blot analysis revealed that p-Stat3 levels were significantly increased in both the soleus and TA muscles of VDR^−/−^ mice compared to those of wild-type mice at both ages examined, while comparable total Stat-3 levels at each time point remained unchanged between WT and V muscles (Fig. 1d, e).

**Fig. 2:**
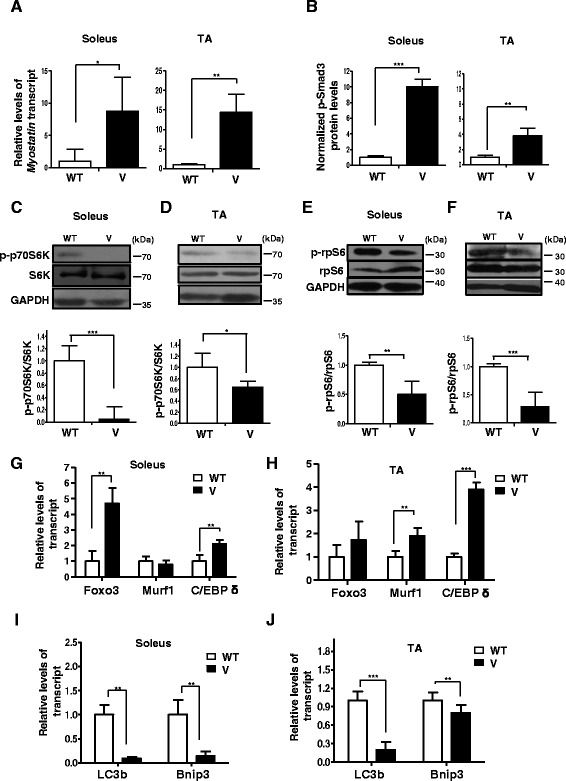
VDR^−/−^ muscles are characterized by an increase in atrophic gene expression, a decline in mTOR pathway components, and a block in autophagic gene expression. **a** The soleus and TA muscles from 6-week-old WT and V mice were assessed for levels of *Myostatin* transcript by qRT-PCR. *Myostatin* transcript levels in the soleus and TA muscles of V mice are normalized to those in WT (*n* = 6) (*p < 0.05). *Myostatin* is upregulated in the soleus and TA muscles of V compared to WT. **b** Graph showing the quantitation of western blot analysis of lysates from dissected soleus muscles (Fig. 1b) and TA muscles (Fig. 1c) from WT and V mice at 6 weeks of age probed for p-Smad3 antibody (*n* = 5) (**p < 0.01, ***p < 0.005). GAPDH serves as a loading control. **c–f** Representative western blot analysis of lysates from dissected soleus muscles (Fig. 1b) and TA muscles (Fig. 1c) from WT and V mice at 6 weeks of age probed for p-p70S6K antibody and S6K antibody (**c, d**) and p-rpS6 antibody and total rpS6 (**e, f**). *Graphs below each blot* show ratios of p-p70S6K to total S6K and p-rpS6 to total rpS6, respectively (*n* = 5) (**p < 0.01, ***p < 0.005). GAPDH serves as a loading control. **g, h** Soleus and TA muscles from 6-week-old WT and TA muscles were assessed for known markers of atrophy, *Foxo3*, *Murf1*, and *C/EBP δ* transcripts by qRT-PCR. While *C/EBP δ* was upregulated in both muscles sets, *Foxo3* and *Murf1* showed differential fiber-type-specific expression (*n* = 5) (**p < 0.01, ***p < 0.005). **i–j** Soleus and TA muscles from 6-week-old WT and TA muscles were assessed for known markers of autophagy, *LC3b* and *Bnip3* transcripts, by qRT-PCR. Both transcripts were downregulated in V muscles compared to WT muscles. All transcript levels in V are normalized to those in WT (*n* = 5) (**p < 0.01, ***p < 0.005)

To investigate the consequence of p-Stat3 signaling on muscle atrophy, we analyzed the expression of *Myostatin*, a gene that has been demonstrated to be a key mediator of atrophy and a target of Stat3 signaling [22], in 8-week-old wild-type and VDR^−/−^ mice. We found that *Myostatin* expression levels were significantly increased in both soleus and TA muscles from VDR^−/−^ mice compared to those of wild-type mice (Fig. 2a).

Further, to assess the consequences of increased Myostatin function, we examined downstream p-Smad signaling in muscles from VDR^−/−^ mice. Myostatin binds to receptor complexes, activin llb, and Alk4/Alk5 receptors that leads to the phosphorylation, activation, and translocation of p-Smad transcription factors into the nucleus [32, 33]. We found that both subsets of muscles in the VDR^−/−^ mice displayed elevated p-Smad3 signaling compared to wild-type mice (Fig. 2b). Both in vitro and in vivo experiments have shown that Myostatin signaling inhibits the activation of the mTOR/p70 S6 kinase (p70S6K) pathway, thereby promoting muscle atrophy in various catabolic conditions [32, 34, 35]. To assess the effects of vitamin D signaling on the mTOR pathway, we analyzed the levels of phosphorylated p70S6K (p-p70S6K) in VDR^−/−^ muscles. We found that both the soleus and TA muscles of VDR^−/−^ mice displayed reduced levels of p-p70S6K in comparison to wild-type mice (Fig. 2c, d). Of the many known substrates of p70S6K, ribosomal protein S6 (rpS6) has been shown to be directly involved, via its phosphorylation in regulating cell size [24]. To examine the effects of p-Stat3 signaling on muscle metabolic activity, we analyzed levels of phosphorylated rpS6 (p-rpS6) from the soleus and TA muscles of 6-week-old VDR^−/−^ mice (Fig. 1). Consistent with reduced p70S6kinase activity, we observed a reduction in p-rpS6 levels compared to wild-type mice of the same age (Fig. 2e, f).

In addition to Myostatin, skeletal muscle atrophy has been demonstrated to be characterized by the expression of markers such as FOXO3, muscle-specific E3 ubiquitin ligase, muscle-RING finger 1 (MuRF1), which is known to mediate the ubiquitin-proteasomal pathway, and C/EBP δ, an effector of Stat3 signaling and a key mediator of the transcriptional activity of Myostatin [22, 36, 37]. While *C/EBP δ* transcripts were observed to be upregulated in both VDR^−/−^ soleus and TA muscles, *Foxo3* and *MuRF1* transcripts displayed a fiber-type-specific pattern of expression, suggesting that slow and fast muscles exhibit differences in the atrophic program induced in the absence of vitamin D signaling (Fig. 2g, h).

Another highly conserved pathway that determines the stability of proteins in the skeletal muscle is autophagic/lysosomal pathway [27]. To evaluate the status of autophagy in VDR^−/−^ muscles, we assessed levels of LC3b and Bnip3 in the soleus and TA muscles of these mice. We found a significant decline in the expression of both LC3b and Bnip3 in both subsets of VDR^−/−^ muscles compared to wild-type muscles, indicating a block in the autophagic process (Fig. 2i, j).

### IL-6 protein levels are increased in the soleus but not in the TA muscles of VDR^−/−^ mice

**Fig. 3:**
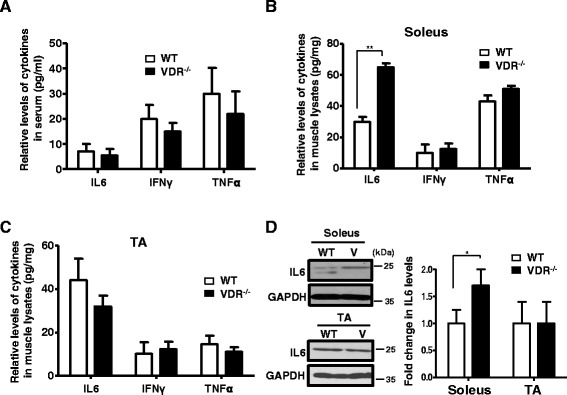
VDR^−/−^ soleus but not TA muscles display increased IL-6 protein levels. **a** Serum IL-6, IFNγ, and TNFα levels were measured by ELISA in triplicate wells from 8-week-old WT and V mice. Graph shows that no significant differences in cytokine levels were observed in the serum from the two sets (*n* = 5). **b**, **c** Cytokine levels in **a** were measured in the soleus (**b**) and TA (**c**) muscle lysates isolated from 8-week-old WT and V mice. Only IL-6 levels were observed to be significantly increased in the soleus muscles of V mice (*n* = 5) (**p < 0.01). Cytokine levels were normalized to tissue weight (mgs). **d** *Top* and *bottom* panels show a representative western blot analysis of lysates from the soleus and TA muscles, respectively, of WT and V mice probed with IL-6 antibody. *Graph to the right* shows the quantitative differences between the two groups. IL-6 protein levels are increased in the soleus but not in the TA muscles of V compared to WT mice. GAPDH serves as the loading control (*n* = 6)

Several pro-inflammatory cytokines (IL-6, IFNγ, and TNFα) are elevated in various catabolic conditions and may trigger muscle wasting by increasing the expression of NF-kB, promoting the expression of other cytokines, or by modulating the activity of regulators of protein synthesis [22, 38]. To investigate the role of cytokines in promoting atrophy in the absence of vitamin D signaling, we estimated the levels of IL-6, IFNγ, and TNFα in the sera and muscle lysates of VDR^−/−^ mice. We used ELISA kits specific for mouse IL-6, IFNγ, and TNFα to obtain standard curves for the respective cytokines (Additional file 1: Figure S1A–C). We did not observe any significant differences in the levels of these cytokines in the sera of VDR^−/−^ mice compared to wild-type mice (Fig. 3a). However, in ELISA assays performed on muscle lysates isolated from VDR^−/−^ and wild-type mice, we observed that IL-6 protein levels and not IFNγ, and TNFα, from VDR^−/−^ soleus muscles were significantly higher than those compared to wild-type soleus muscles (Fig. 3b). On the other hand, IL-6 protein levels were not enhanced in the TA muscles of VDR^−/−^ mice compared to those of wild-type mice (Fig. 3c). Corroborating these results, we found an increase in IL-6 protein levels in the soleus muscles of VDR^−/−^ mice using western blot analysis (Fig. 3d). These results are consistent with the reports on the preferential activation of Stat3 by IL-6 and the increase in IL-6 gene expression in slow muscles during exercise [39–42] and suggest that elevated levels of IL-6 protein could contribute to the increase in p-Stat3 signaling in the soleus, while presumably other upstream factors promote increase p-Stat3 in the TA muscles of VDR^−/−^ mice.

### VDR^−/−^ SCs are similar to wild-type SCs in numbers and Myostatin expression but display hallmarks of atrophy upon differentiation in culture

**Fig. 4:**
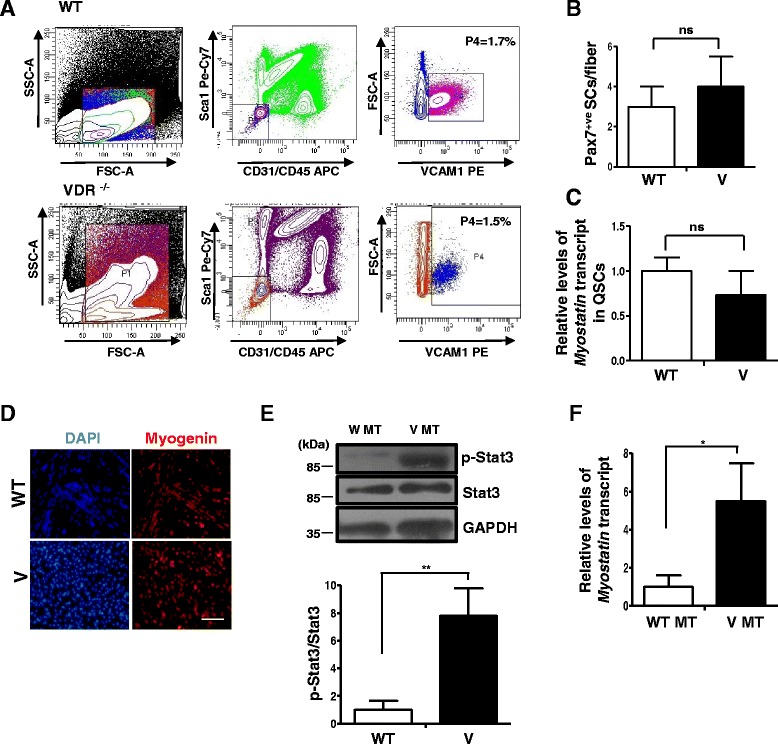
VDR^−/−^ SCs do not differ in number or *Myostatin* expression from wild-type SCs but display hallmarks of atrophy upon differentiation in culture. **a** SC numbers were quantified by FACS analysis of mononuclear cells from hind limb muscles of WT and V mice. SCs are shown in *purple* (WT) and *blue* (V) in these representative FACS plots. In three replicates, the percentage of SCs in total mononuclear cells in V muscles was not significantly different (1.5%) compared to that in WT muscles (1.7%) (*n* = 3 mice per genotype were pooled per experiment, *N* = 3). **b** Single myofibers were obtained from WT and V muscles and stained for Pax7 to assess SC numbers per fiber. There were no statistically significant differences in the number of SCs per fiber between the two strains. Fibers were obtained from three mice per genotype. **c** FACS-purified quiescent SCs from WT and V mice muscles were assessed for levels of *Myostatin* expression by qRT-PCR. *Myostatin* transcript levels in V are normalized to WT. Quiescent SCs from each genotype represent triplicate experiments of pooled RNA from three mice for each experiment (*n* = 3 mice per genotype were pooled per experiment, *N* = 3). **d** FACS-sorted SCs from WT and V hind limb muscles were induced to differentiate for 2 days and stained for Myogenin. **e** Western blot analysis of lysates from differentiated SC cultures from WT (WT MT) and V (V MT) mice were probed with p-Stat3 and total Stat3 antibodies. Graph below shows a significant upregulation in p-Stat3 levels in V MT cultures compared to those in WT MT (*n* = 3 mice per genotype were pooled per experiment, *N* = 3). (**p < 0.01). **f** Myotubes from WT and V muscles were assessed for levels *Myostatin* expression by qRT-PCR. *Myostatin* is upregulated in V myotubes compared to WT myotubes (*p < 0.05)

To investigate the effects of VDR-deficient status on the SC compartment in skeletal muscles, we isolated SCs by FACS as previously described [29] from the hind limb muscles of VDR^−/−^ mice and wild-type mice (Fig. 4a). We did not observe any differences in the relative proportion of SCs in bulk muscles isolated from wild-type mice and VDR^−/−^ mice (Fig. 4a, third panel in the top and bottom rows, respectively). We then isolated single muscle fibers from wild-type and VDR^−/−^ muscles and fixed them immediately after isolation. We stained fibers with a Pax7 antibody that identifies quiescent SCs and found no significant differences in SC numbers between wild-type and VDR^−/−^ fibers, consistent with the FACS results (Fig. 4b). These results suggest that VDR signaling does not influence specification and maintenance of SC numbers and that the muscle atrophy observed in VDR^−/−^ mice is not due to a deficit in SC numbers in these muscles.

Since expression levels of *Myostatin* were found to be significantly upregulated in both muscle sets in the VDR^−/−^ mice and *Myostatin* is a key regulator of self-renewal in SCs, we wanted to investigate whether changes in *Myostatin* expression in VDR^−/−^ SCs accompany atrophy in VDR^−/−^ muscles. Unlike in the fibers, quantitative RT-PCR analysis revealed no significant differences in levels of *Myostatin* expression between VDR^−/−^ and wild-type SCs (Fig. 4c). This suggests that a role for *Myostatin* in promoting atrophy in VDR^−/−^ muscles is predominantly confined to the regulation of its expression within fibers rather than the SC compartment.

Since the molecular changes described thus far were observed in a global knockout of VDR, raising the possibility of our observations emerging as secondary consequences to metabolic perturbations in the muscle niche, we cultured FACS-isolated SCs in vitro and differentiated them for 2 days to obtain an independent validation of our results (Fig. 4d). We observed that SC progeny from both VDR^−/−^ and wild-type muscles displayed Myogenin expression upon differentiation in culture (Fig. 4d). A western blot analysis on cell lysates from VDR^−/−^ myotubes revealed elevated levels of p-Stat3 compared to wild-type myotubes (Fig. 4e). Further, qRT-PCR analysis revealed that VDR^−/−^ myotubes displayed significantly elevated *Myostatin* expression compared to wild-type myotubes (Fig. 4f). Taken together, these results suggest an intrinsic deficit in the SC compartment that could impair their ability to contribute to postnatal muscle growth in the absence of vitamin D signaling.

### Inhibition of Stat3 signaling ameliorates muscle mass loss in slow but not fast muscles of VDR^−/−^ mice

**Fig. 5:**
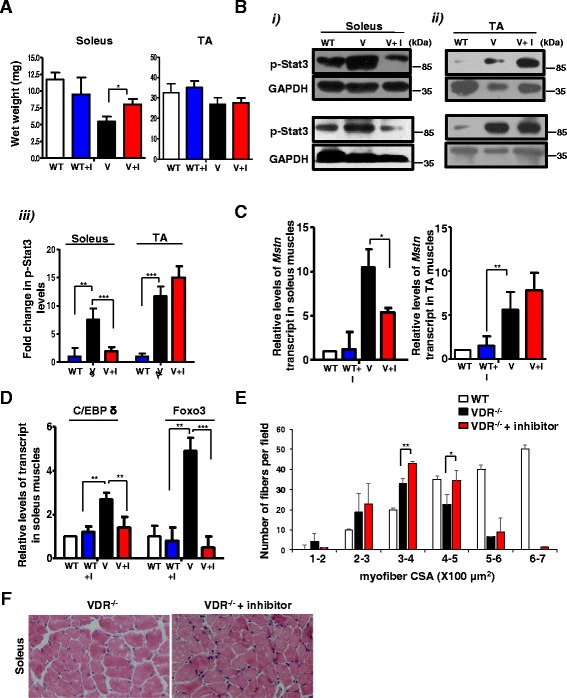
Inhibition of Stat3 activity ameliorates muscle mass loss in VDR^−/−^ soleus muscles. **a** Average weights of the soleus (*left graph*) and TA muscles (*right graph*) from WT and paired V mice that were injected with either the diluent (WT and V, respectively) or C188-9 (WT + I and V + I, respectively) after 2 weeks of treatment (*n* = 5 mice/group, *N* = 3 independent experiments). Soleus muscles from V + I mice show a significant increase in weight compared to those from V mice injected with diluent (*p < 0.05). **b** (*i*) and (*ii*) Representative western blot analysis of lysates in duplicate from independent experiments from soleus (*i)*, *top* and *bottom left* panels, and TA muscles (*ii*), *top* and *bottom right* panels, from WT, V, and V + I mice. Blots were probed with p-Stat3 antibody. Fold differences in p-Stat3 levels were calculated by normalizing intensities with respective GAPDH values (*n* = 5 mice/group, *N* = 3 independent experiments). (*iii*) Graph shows quantitative analyses of replicative blots of levels of p-Stat3 protein in the TA and soleus muscles after inhibitor treatment (**p < 0.01, ***p < 0.005). A detectable decrease in p-Stat3 levels were observed in soleuses of V + I mice, but not in the TA muscles. **c** The soleus muscles (*left graph*) and TA muscles (*right graph*) from paired V and V + I mice and WT + I mice and were assessed for *Myostatin* transcript levels by qRT-PCR. There was a significant decline in *Myostatin* expression in V + I soleus muscles compared to control V muscles, but not in the TA subset of inhibitor-treated V mice (*p < 0.05). **d** The soleus muscles from same group as **c** were assessed for *C/EBP δ* and *Foxo3* expression by qRT-PCR. There was a reduction in *C/EBP δ* and *Foxo3* expression in V + I soleus muscles compared to control V muscles (**p < 0.01, ***p < 0.005). **e–f** Cryosections of the soleus muscles isolated from V (*top panel*) and V + I (*bottom panel*) mice were stained for hematoxylin and eosin. Individual fibers were evaluated for cross-sectional area (CSA) in both groups. A minimum of 800 fibers were counted per section from soleuses in three independent experiments for each genotype. V + I soleus fibers show reduced number of smaller fibers of sizes 100–200 μm and a greater homogeneity in fibers with sizes 300–400 μm (S.D = 1.414 for V + I compared to 5.2 for V) (*p < 0.05). There was also a significant increase in fibers with sizes from 400 to 500 μm. *Scale bars* represent 100 μm

To determine whether a pharmacological inhibition of Stat3 signaling can be used to block muscle wasting observed in the VDR^−/−^ mice, we injected four-and-a-half-week-old VDR^−/−^ mice that had been paired for their body weights or wild-type mice with C188-9, a small molecule inhibitor of Stat3 or the diluent, with 5DW for 2 weeks. We found that C188-9-treated VDR^−/−^ mice displayed a significant increase in the wet weight of soleus (45% over vehicle-treated VDR^−/−^ mice), but not the TA muscles after treatment, although there were no significant changes in the total body weight of the mice (Fig. 5a). However, the soleus muscles from wild-type mice injected with C188-9 did not show any differences in muscle weight from the vehicle-treated controls (Fig. 5a). Further analysis revealed that while C188-9 treatment did not decrease the level of p-Stat3 in wild-type soleus and TA muscles (Additional file 2: Figure S2A), VDR^−/−^ mice injected with C188-9 displayed a significant reduction in p-Stat3 levels in the soleus (Fig. 5b (i), top and bottom panels, and Fig. 5b (iii)) but not in the TA muscles (Fig. 5b (ii), top and bottom panels*,* and Fig. 5b (iii)). To investigate the status of Stat3 signaling in other muscles in inhibitor-treated animals, we isolated the quadriceps muscle, which like the TA has a predominance of type ll fibers, and found that while there was an increase in p-Stat3 levels in VDR^−/−^ quadriceps compared to the wild-type, C188-9 treatment did not ameliorate muscle mass loss or decrease the level of p-Stat3 in quadriceps muscles compared to vehicle-injected control VDR^−/−^ mice (Additional file 3: Figure S3A–C). To evaluate the consequences of amelioration of the atrophic response due to decreased p-Stat3 levels on changes in gene expression, we assessed levels of *Myostatin* in the soleus and TA muscles of C188-9-treated and vehicle-treated VDR^−/−^ and wild-type mice. We found that there was a significant decrease in *Myostatin* expression in the soleus muscles of C188-9 treated VDR^−/−^ mice and not in the TA muscles compared to vehicle-injected controls, consistent with an amelioration of atrophy in the soleus muscles (Fig. 5c). Similarly, *C/EBP δ* and *Foxo3* expressions that were previously found to be upregulated in the soleus muscles of VDR^−/−^ mice also declined upon inhibitor treatment (Fig. 5d). However, wild-type mice injected with C188-9 did not show any relative differences in *Myostatin* expression from vehicle-injected controls (Fig. 5c). These results suggest that compared to slow muscles, the fast muscles are less responsive to the inhibition of Stat3 signaling.

To examine the consequences of Stat3 pharmacological inhibition on gross morphometry of the soleus muscles, we stained cryosections of the soleus muscles isolated from these mice with hematoxylin and eosin (Fig. 5f, left and right panels for control and treated VDR^−/−^ mice, respectively). While we observed a wide variation in the size of fibers in VDR^−/−^ mice consistent with earlier observations [13], VDR^−/−^ mice injected with C188-9 displayed a reduced number of smaller fibers (100–200 μm) compared to vehicle-injected controls (Fig. 5e). Additionally, we observed a greater homogeneity in the larger fibers (300–400 μm) in VDR^−/−^ mice injected with C188-9 (S.D ± 1.414, *n* = 5 mice in three independent experiments), compared to vehicle-injected controls (S.D ± 5.2, *n* = 5 in three independent experiments), and a significant increase in fibers with sizes from 400 to 500 μm (Fig. 5e).

### Amelioration of muscle mass in the soleus of VDR^−/−^ mice is accompanied by a decline in Myostatin function and restoration of mTOR signaling

**Fig. 6:**
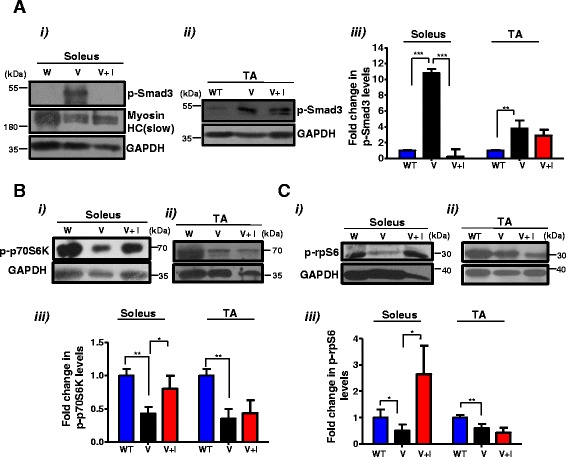
Inhibition of Stat3 activity ameliorates the changes in levels of mTOR components observed in VDR^−/−^ soleus muscles. **a–c** (*i*) and (*ii*) Representative western blot analysis of lysates from **b** were probed with p-Smad3 (**a**), p-p70S6K (**b**), and p-rpS6 (**c**) antibodies. The p-Smad3 blot was also probed with MHC (slow) as a representative example to mark the identity of the lanes. **a–c**, (*iii*) Graphs show fold differences in p-Smad3, p-p70S6K, and p-rpS6, respectively, that were calculated by normalizing intensities with respective GAPDH values (*n* = 5 mice/group, *N* = 3 independent experiments) (*p < 0.05, **p < 0.01). While p-Smad3 levels were reduced in the soleus muscles of inhibitor-treated V mice, p-p70S6K and p-rpS6 levels were increased

Since we observed an amelioration of muscle mass in soleus muscles and a decline in *Myostatin* expression in inhibitor-treated VDR^−/−^ mice, we examined downstream p-Smad signaling as a consequence of decreased Myostatin function. We found that VDR^−/−^ mice injected with C188-9 displayed a significant reduction in p-Smad3 levels in the soleus, but not in the TA muscles (Fig. 6a (i–iii)). As a representative example to confirm the identity of the lanes, we also probed these blots with an anti-Myosin heavy chain (HC) antibody specific to slow muscles. Interestingly, we found that VDR^−/−^ soleus muscles displayed reduced levels of slow Myosin HC compared to those of the wild-type, suggesting that there could be a decline in sarcomere integrity in the absence of VDR signaling (Fig. 6a (i)).

To further evaluate the consequences of p-Stat3 inhibition and reduced Myostatin signaling on the mTOR/p70S6K pathway, we examined the status of p-p70S6k and its downstream effector, p-rpS6 (Fig. 6b, c). We found that p-p70S6K levels increased in VDR^−/−^ mice injected with C188-9 in the soleus but not in the TA muscles (Fig. 6b (i–iii)). Similarly, p-rpS6 levels were also restored in the soleus muscles of inhibitor-treated VDR^−/−^ mice, an effect that was not observed in the VDR^−/−^ TA muscles or in the wild-type mice injected with C188-9 (Fig. 6c (i–iii) and Additional file 2: Figure S2B).

### Differential response of fiber types to Stat3 pharmacological inhibition could be due to preferential fiber type p-Stat5 levels and its dependence on vitamin D signaling

**Fig. 7:**
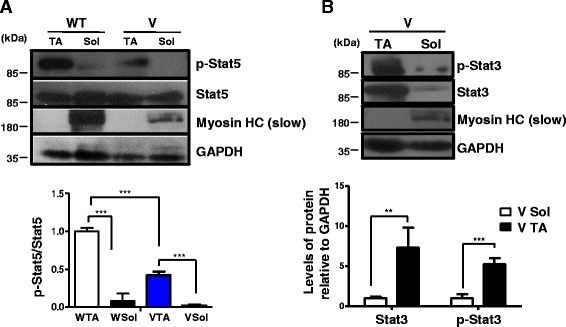
Different levels of Stat3 expression and activity in VDR^−/−^ muscle subsets amenable to pharmacological inhibition could be linked to preferential fiber type expression of p-Stat5 and its dependence on vitamin D signaling. **a** Representative western blot analysis of lysates from the soleus and TA muscles isolated from 6-week-old WT and V mice were probed with p-Stat5 and total Stat5 antibodies. Graph below show the fold differences in levels of p-Stat5 to total Stat5 protein that was calculated after normalizing band intensities with respective GAPDH values (*n* = 5 mice/group, ***p < 0.005). Levels of p-Stat5 are significantly higher in the TA muscles than those in the soleus muscles and are downregulated in the absence of VDR signaling. **b** Western blot analysis of lysates from the soleus and TA muscles of V mice were probed with p-Stat3 and total Stat3 antibodies. Graph below shows the fold differences in levels of p-Stat3 and total Stat3 after normalizing band intensities with respective GAPDH values (*n* = 5, ***p < 0.005, **p < 0.01). Blots were probed with MHC (slow) to mark the identity of the lanes. The TA muscles express significantly higher levels of p-Stat3 and total Stat3 compared to the soleus muscles from V mice

To address our observations on the differential responses of the soleus and the TA muscles to Stat3 pharmacological inhibition, we hypothesized (a) the presence of a key regulator that displays preferential fiber-type-specific function and (b) whose absence results in quantitatively higher levels of p-Stat3 relative to the other subset of muscles, thereby rendering the pharmacological intervention ineffective. Previous studies have shown that in the skeletal muscle, Stat5 functions to repress Stat3 expression, such that a muscle-specific knockout of Stat5 results in the upregulation of Stat3 mRNA [43]. To check the expression and activity of Stat5 in slow versus fast muscles, we performed a western blot analysis of lysates isolated from wild-type soleus and TA muscles. We found that p-Stat5 was expressed at significantly higher levels in the TA than in the soleus muscles (Fig. 7a). A similar observation was made between muscle subsets isolated from VDR^−/−^ mice (Fig. 7a). Importantly, we found that p-Stat5 levels were downregulated in a quantitative comparison between the TA muscles of VDR^−/−^ and wild-type mice, indicating that VDR signaling promotes Stat5 function (Fig. 7a).

To address the possibility that reduced levels of p-Stat5 because of absent VDR signaling might account for the higher levels of p-Stat3 in the TA muscles rather than in the soleus muscles of VDR^−/−^ mice, we compared active and total Stat3 levels in the TA and soleus muscles of VDR^−/−^ mice (Fig. 7b). We observed quantitatively higher levels of not only p-Stat3 but also total Stat3 levels in the TA muscles of VDR^−/−^ mice compared to those in the soleus muscles (Fig. 7b). Thus, Stat5 activity imposes an additional level of regulation on the Stat3-Myostatin axis in fast muscles, such that upon its downregulation during reduced vitamin D signaling, p-Stat3 levels increase several folds in fast muscles compared to slow muscles, thereby impeding effective pharmacological inhibition.

**Fig. 8:**
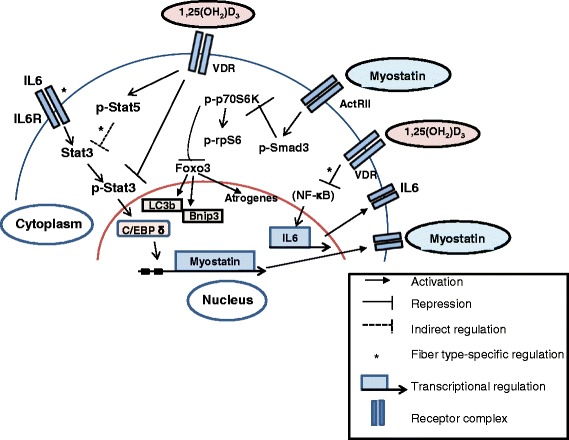
Working model representing the proposed signaling pathways induced by vitamin D signaling in regulating skeletal muscle mass. Active vitamin D bound to vitamin D receptors maintains proteostasis by inhibiting p-Stat3 signaling and downstream C/EBP δ activity, thereby inhibiting the production of Myostatin. In fast muscles, active vitamin D additionally promotes p-Stat5 expression that could function to inhibit Stat3 expression and function. In the absence of vitamin D signaling, increased levels of Myostatin induces p-Smad3 signaling, which in turn inhibits components of the mTOR pathway, p-p70S6 kinase, and p-rpS6. Translocation of active FOXO3 into the nucleus due to reduced mTOR pathway signaling can upregulate the expression of various atrogenes. Paradoxically, autophagy regulators and targets of FOXO3 signaling, LC3b and Bnip3, display decreased expression, suggesting a block in the autophagic process and possibly an inhibition of recruitment of FOXO3 to the promoters of these genes. Additionally, active vitamin D reduces IL-6 production in slow muscles, possibly through the inhibition of the NF-kb complex that further attenuates p-Stat3 signaling

Taken together, our results suggest a working model whereby vitamin D maintains muscle mass by inhibiting Stat3 signaling and Myostatin expression and function, thereby sustaining mTOR signaling and promoting protein synthesis. In fast muscles, vitamin D promotes Stat5 function that could further repress Stat3, while in slow muscles, vitamin D reduces IL-6 cytokine levels as a mechanism to suppress Stat3 signaling (Fig. 8).

## Discussion

Our results demonstrate that VDR^−/−^ muscles display elevated Stat3 signaling and increased *Myostatin* expression. Increased Myostatin signaling induces p-Smad3 activity that is known to suppress mTOR signaling. Consequently, we observed a decline in p-p70S6K and p-rpS6 levels in VDR^−/−^ muscles. More importantly, pharmacological inhibition of Stat3 function decreased p-Stat3, *Myostatin* expression, and p-Smad3 levels, while restoring p-p70S6K and p-rpS6 levels resulting in an amelioration of muscle mass loss, an effect that was observed in the soleus muscles of VDR^−/−^ mice.

### Inhibition of Stat3 function ameliorates the loss of muscle mass in slow muscles

Although fiber type sensitivities to atrophy vary depending on the atrophic stimuli, VDR^−/−^ muscles have been reported to display smaller fiber diameter, irrespective of the fiber type [13, 44]. In keeping with this observation, p-Stat3 signaling was found to be elevated in both fast (TA and quadriceps) and slow (soleus) muscles of VDR^−/−^ mice suggesting a common contributory factor for promoting the atrophic process in both fiber types. However, we observed that the soleus and not the TA or quadriceps muscles responded to the inhibition of Stat3 function. Our results confirmed the hypothesis that the relative levels of p-Stat3 in VDR^−/−^ fast muscles could be higher than those in the soleus muscles, resulting in the wasting process being more effectively blocked by the pharmacological inhibitor in the soleus muscles alone (Fig. 7b). Since Stat3 plays a crucial role in cell proliferation and motility during early development and SCs are rapidly dividing until 3 weeks after birth to contribute to myofiber growth, we chose to initiate the inhibition of Stat3 function after this critical period to avoid interfering with myogenic activity during early postnatal development [45, 46]. Additionally, the dose and extent of treatment was the same as that used in a previous study to inhibit loss of muscle mass observed in models of muscle wasting in mice [22]. This was done to ensure effective inhibition of Stat3 function without confounding effects, thus making it unlikely that the observations made in this study were restricted to the format of the treatment regimen alone. Our results propose a working model wherein high levels of p-Stat3 in VDR^−/−^ fast muscles could very likely be the result of dysregulated expression of Stat5 in the absence of VDR signaling (Fig. 7a). Corroborating this evidence, Stat5 has been demonstrated to be induced after treatment with 1α,25-dihydroxyvitamin D_3_ in osteoclast cells [47]. Thus, in the absence of vitamin D signaling, activated Stat3 levels increase due to a reduction in Stat5 function. Additionally, Stat5 functions to repress genes associated with type 1 fibers, a report that is consistent with our finding that Stat5 function is quantitatively lower in type 1 fibers (Fig. 7a) [43]. Together, these results suggest that the Stat5-Stat3 regulatory axis is critical in type II fibers, serving to suppress the expression of genes associated with slow fibers and posing as an additional level of regulation of p-Stat3 activity in fast fibers. As such, a combinatorial treatment ensuring increased Stat5 function and decreased Stat3 signaling might contribute to increased weights in muscles with type ll fiber predominance in vitamin D-lacking muscles. Another implication of the intriguing observation that p-Stat3 inhibition ameliorated muscle mass only in the soleus (Fig. 5) suggests the possibility of additional mechanisms that function to restrict p-Stat3 signaling in fast muscles. The downregulation of these modulators that are (a) fast fiber type specific, (b) dependent on vitamin D signaling, and (c) that impinge on the Stat3-Myostatin axis could render fast muscles more susceptible to atrophy than slow muscles, a phenomenon that is observed in vitamin D deficiencies in humans [20]. In this study, we suggest that p-Stat5 signaling could be one such modulator that contributes to the fiber-type-specific effects of vitamin D signaling in wild-type muscles, such that fast muscles that display relatively higher levels of p-Stat5 signaling are more responsive to the growth-promoting effects of vitamin D function. Conversely, slow muscles would be susceptible to a lesser extent to modulations in p-Stat5 activity as a consequence of altered vitamin D signaling. Thus, despite displaying generalized fiber atrophy, the VDR^−/−^ mouse model might offer insights into identifying mechanisms that promote fiber-type-specific atrophy during vitamin D deficiency.

### Key regulators of muscle mass are altered in the absence of vitamin D signaling

Studies examining the effects of vitamin D on myotube formation in C2C12 cell cultures have revealed an increase in myotube size following treatment with 1α,25(OH)_2_D_3_ in a manner independent of its effects on myoblast differentiation [48, 49]. Our results corroborate this finding that in the absence of active vitamin D signaling, key regulators of cell size and tissue mass such as Myostatin, p70S6K, and p-rpS6 are altered. Several possible mechanisms offer clues as to how p-Stat3 might induce muscle wasting in VDR^−/−^ mice. Firstly, VDR^−/−^ muscles in response to elevated Stat3 signaling display a reduction in p-rpS6 levels (Fig. 2), a key regulator of cell growth which upon inhibition mirrors suppression of mTOR function in the regulation of cell size [24, 50]. A similar pattern of molecular expression has been reported in a recent study to characterize muscle atrophy following burn injuries, wherein human skeletal muscle SCs incubated in burn serum displayed elevated Stat3 levels and decreased p70S6K and p-rpS6 protein levels [51]. In rpS6^P−/−^ mice, it was shown that although mouse embryonic fibroblasts (MEFs) derived from these mice were significantly smaller than rpS6^P+/+^ MEFs, there was no corresponding decline in global protein synthesis, suggesting that the atrophy observed in VDR^−/−^ mice cannot be explained solely by a decline in protein synthesis [24]. Whether other cellular components are affected by decreased rpS6 activity have yet to be investigated. In relation to the role of rpS6 in skeletal muscles, it has been demonstrated that the soleus muscles from the rpS6^P−/−^ mice displayed a decrease in muscle mass due to a diminished abundance of contractile proteins and reduced energy content, thereby supporting the function of rpS6 as a potential mediator of the effects of vitamin D signaling on muscle mass [52]. Of the contractile proteins downregulated in rpS6^P−/−^ soleus muscles, Myosin HC was also observed to be reduced in VDR^−/−^ soleus muscles, further corroborating the role of rpS6 downstream of VDR signaling [52] (Figs. 6 and 7). Secondly, we observed a significant upregulation of *Myostatin* expression, a potent negative regulator of muscle mass in the VDR^−/−^ mice, in sets of muscles that differ in their fiber type content (Fig. 2a). Increased levels of Myostatin have been found in aging subjects and in various diseases that are characterized by muscle wasting [53]. It has been demonstrated that p-Stat3 elevates *Myostatin* expression through the modulation of CCAAT/enhancer-binding protein (CEBP) δ and that the Myostatin promoter bears multiple binding sites for CEBP δ [22]. Indeed, in our studies, we observed an upregulation in CEBP δ transcript levels in both the soleus and TA muscles of VDR^−/−^ mice (Fig. 2g–h). Additionally, treatment of C2C12 myoblasts with 1α,25(OH)_2_D_3_ downregulated *Myostatin* expression, suggesting a causal link between vitamin D signaling and a key regulator of muscle mass [48]. Moreover, we also see an upregulation in *Foxo3* expression, which is known to cause a dramatic atrophy in myotubes and muscle fibers through its effects on autophagy, such that a knockdown of LC3, a protein involved in autophagosome formation, partially ameliorates Foxo3-mediated muscle mass loss (Fig. 2g) [27]. However, the upregulation of *Foxo3* expression was more apparent in the soleus muscles rather than in the TA muscles of the VDR^−/−^ mice, an observation that stands in contrast to the study on Foxo3 in mediating glycolytic (type 2) rather than in oxidative (type 1) fiber atrophy [54]. These results might be more explained by the stimuli that initiate the atrophy process in various muscle wasting models. For example, VDR^−/−^ mice are known to display impaired motor functions and type 1 fiber atrophy is more sensitive to neuromuscular perturbations [31, 55]. As such, the upregulation of *Foxo3* mRNA in the soleus might be the outcome of a combination of metabolic and neural abnormalities. Surprisingly, an analysis of the expression pattern of key autophagic genes, LC3b and Bnip3, that mediate the effects of Foxo3 on autophagy was found to be downregulated in VDR^−/−^ muscles, suggesting a block in the autophagic process (Fig. 2i–j). This could lead to the accumulation of damaged and dysfunctional organelles that impair tissue function, as observed in the Atg7^−/−^ mice that lack the unique E1 enzyme of the autophagic machinery [56]. The block in autophagy causes disorganized sarcomeres, myofiber degeneration, and accumulation of polyubiquitinated proteins that manifest in the form of severe muscle weakness and myopathy in the Atg7^−/−^ mice [56]. However, detailed molecular analyses such as the visualization of LC3-positive granules and p62 protein that recruits autophagic vesicles to ubiquitylated mitochondrial proteins as well as mitochondrial network remodeling are critical in distinguishing between a stalled autophagic process and increased turnover rates of autophagic markers that prevent their detection. Nevertheless, our initial results are indicative of a dysfunctional autophagic process in VDR^−/−^ muscles that could exacerbate muscle wasting.

### Effects of the absence of vitamin D signaling on SC numbers and Myostatin expression

Several recent reports have addressed the molecular changes taking place in the stem cell compartment accompanying various forms of atrophy [57]. Studies examining the changes in SC compartment in muscle wasting conditions such as aging and hind limb suspension have reported variable results from a reduction to no differences in SC numbers under these conditions [58–60]. In our study, we observed that VDR^−/−^ muscles do not possess a stem cell deficit compared to wild-type muscles, underscoring the need for a qualitative assessment of SC functionality, rather than quantitative parameters (Fig. 4a, b). These results indicate that vitamin D signaling does not play a decisive role in determining the numbers of SCs during development. We also observed that *Myostatin* mRNA levels were unaltered in quiescent VDR^−/−^ muscles, unlike their expression in fibers, indicating a differential regulation in fiber and SC compartments within the atrophying VDR^−/−^ muscles (Fig. 4c). Indeed, *Myostatin* mRNA levels were reported to be unaltered in human muscle precursor cells upon 1α,25(OH)_2_D_3_ treatment [14], suggesting that the modulation of *Myostatin* in VDR^−/−^ muscles might be confined to the fiber alone.

## Conclusions

The purpose of our study was to determine the molecular mechanisms underlying the loss of skeletal muscle mass in the absence of vitamin D signaling. In the absence of vitamin D signaling, both the soleus and the TA muscles that represent examples of slow and fast subsets of muscles, respectively, display an increase in p-Stat3 levels and *Myostatin* expression. Consequently, p-Smad3 signaling is induced that suppresses p-p70S6K and p-rpS6 levels. In addition to *Myostatin*, we also observed an increase in other atrogenes and a block in autophagy in VDR^−/−^ muscles. Investigations into the upstream regulation of p-Stat3 revealed that IL-6 protein expression, albeit unaltered in the sera, was increased in the soleus but not in the TA muscles in the absence of vitamin D signaling. To understand the role of SCs in the atrophy accompanying the absence of VDR signaling, we found that SC numbers in VDR^−/−^ mice muscles were no different from wild-type, suggesting that active vitamin D signaling is not a prerequisite for determining SC numbers in the skeletal muscle. However, SC progeny that were induced to differentiate in culture displayed an increase in p-Stat3 levels and *Myostatin* expression indicative of a cell-autonomous defect that could impair their ability to participate in postnatal muscle growth or regeneration. Concomitant to the inhibition of p-Stat3, Myostatin-mediated suppression of mTOR signaling was alleviated as evidenced by a restoration in the levels of p-p70S6K and p-rpS6 activity in slow muscles. However, the dysregulated function of Stat5 in the absence of VDR signaling could be responsible for increasingly higher levels of Stat3 expression and function, thereby impeding effective pharmacological inhibition in fast muscles.

## Additional files

Additional file 1: Figure S1. Cytokine standard curves. (PDF 307 kb)
Additional file 2: Figure S2. Treatment with Stat3 inhibitor does not alter levels of p-Stat3 and p-rpS6 in wild-type mice. (PDF 21 kb)
Additional file 3: Figure S3. Quadriceps muscles from inhibitor-treated VDR^−/−^ mice do not show an amelioration of muscle mass or a decrease in p-Stat3 levels. (PDF 18 kb)

## Abbreviations

5DW: 5% Dextrose in water
mTOR: Mammalian target of rapamycin
p70S6K: Ribosomal protein S6 kinase
rpS6: Ribosomal protein S6
SC: Satellite cell
Stat3: Signal transducer and activator of transcription 3
Stat5: Signal transducer and activator of transcription 5
TA: Tibialis anterior
VDR: Vitamin D receptor
