# Supplementary figures and images for: Inhibition of Stat3 signaling ameliorates atrophy of the soleus muscles in mice lacking the vitamin D receptor
Source: Skelet Muscle. 2017 Jan 25;7:2. doi: 10.1186/s13395-017-0121-2 (PMC5264327; doi:10.1186/s13395-017-0121-2)

A

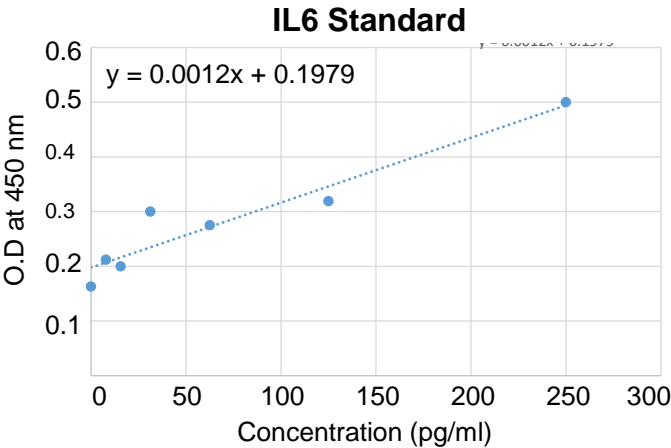

B

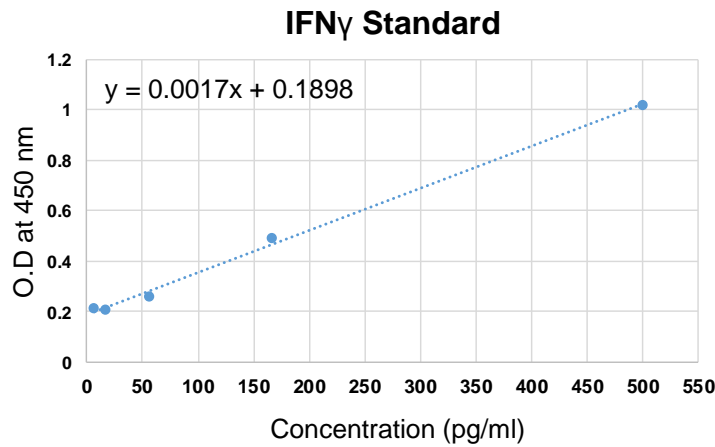

C

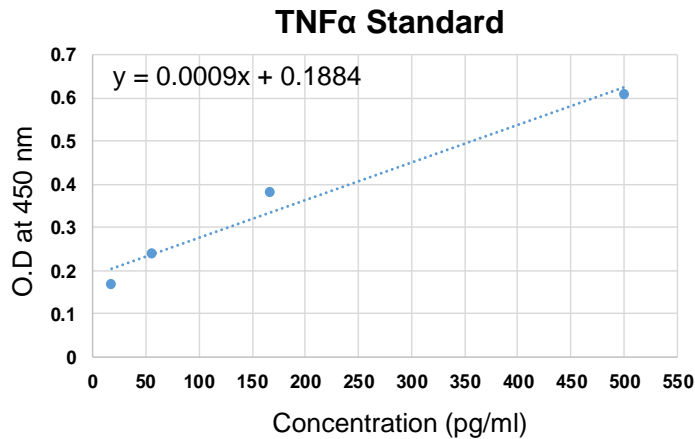

Supplement: Additional file 1: Figure S1. — Cytokine standard curves. (PDF 307 kb) [file 13395_2017_121_MOESM1_ESM.pdf]

Figure S2

A

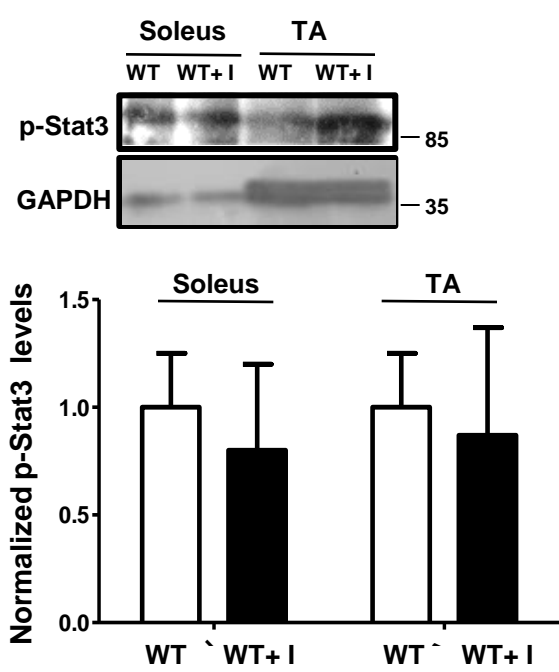

B

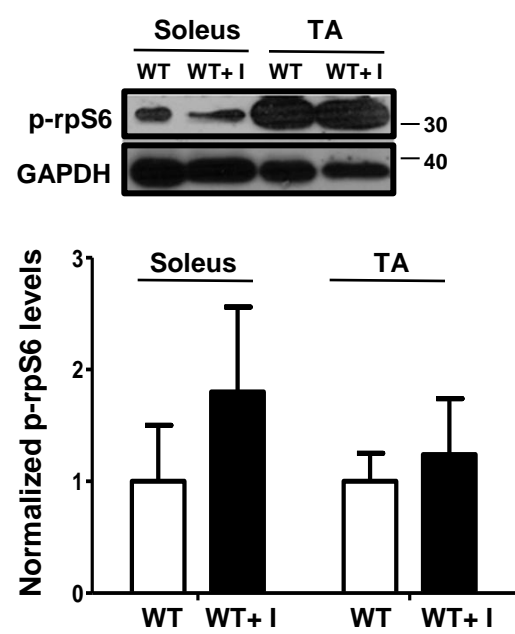

Supplement: Additional file 2: Figure S2. — Treatment with Stat3 inhibitor does not alter levels of p-Stat3 and p-rpS6 in wild-type mice. (PDF 21 kb) [file 13395_2017_121_MOESM2_ESM.pdf]

Figure S3

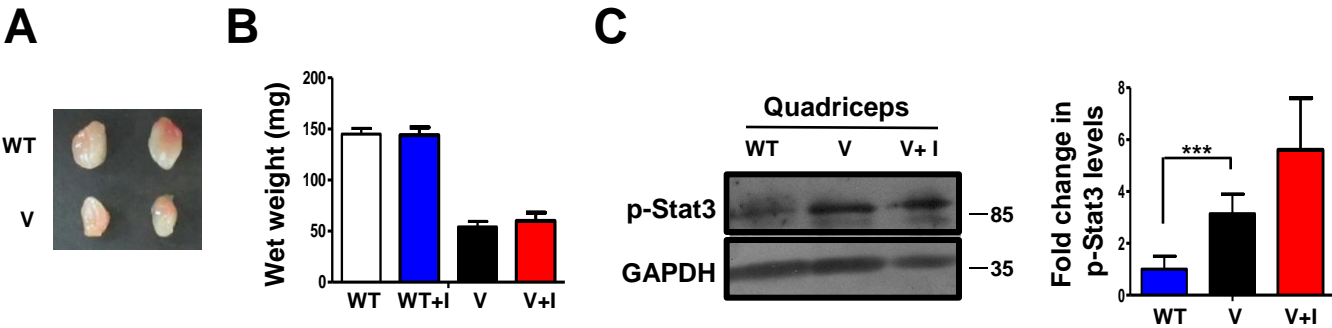

Supplement: Additional file 3: Figure S3. — Quadriceps muscles from inhibitor-treated VDR−/− mice do not show an amelioration of muscle mass or a decrease in p-Stat3 levels. (PDF 18 kb) [file 13395_2017_121_MOESM3_ESM.pdf]
